# A New Apparatus for Standardized Rat Kidney Biopsy

**DOI:** 10.1371/journal.pone.0115368

**Published:** 2014-12-15

**Authors:** Holger Schirutschke, Lars Gladrow, Christian Norkus, Simon Paul Parmentier, Bernd Hohenstein, Christian P. M. Hugo

**Affiliations:** 1 Division of Nephrology, Department of Internal Medicine III, University Hospital Carl Gustav Carus at the Technische Universität Dresden, Dresden, Germany; 2 Institute for Mechanical Engineering, Technische Universität Dresden, Dresden, Germany; University Medical Center Utrecht, The Netherlands

## Abstract

Survival biopsies are frequently applied in rat kidney disease models, but several drawbacks such as surgical kidney trauma, bleeding risk and variable loss of kidney tissue are still unsolved. Therefore, we developed an easy-to-use core biopsy instrument and evaluated whether two consecutive kidney biopsies within the same kidney can be carried out in a standardized manner. On day 0, 18 Lewis rats underwent a right nephrectomy and 9 of these rats a subsequent first biopsy of the left kidney (Bx group). 9 control rats had a sham biopsy of the left kidney (Ctrl group). On day 7, a second kidney biopsy/sham biopsy was performed. On day 42, all animals were sacrificed and their kidneys were removed for histology. Biopsy cylinders contained 57±28 glomeruli per transversal section, representing an adequate sample size. PAS staining showed that the biopsy depth was limited to the renal cortex whereas surgical tissue damage was limited to the area immediately adjacent to the taken biopsy cylinder. On day 42, the reduction of functional renal mass after two biopsies was only 5.2% and no differences of body weight, blood pressure, proteinuria, serum creatinine, glomerulosclerosis, interstitial fibrosis or number of ED-1 positive macrophages were found between both groups. In summary, our apparatus offers a safe method to perform repetitive kidney biopsies with minimal trauma and sufficient sample size and quality even in experimental disease models restricted to one single kidney.

## Introduction

Despite the existence of a wide variety of kidney disease models in the rat, no standardized method for rat kidney biopsies has been developed. However, survival rat kidney biopsies are frequently applied to save animal numbers. Biopsies are performed during an open surgical procedure, removing a variable volume close to that of a 1/4 or 1/3 nephrectomy. This approach may lead to a series of unwanted side effects, especially when used in single kidney disease models such as kidney transplantation, renal artery perfusion, ureteral ligation, accelerated models with unilateral nephrectomy or during experiments with enhanced bleeding disposition. Large wound areas (due to the freehand usage of scalpels or shavers) can cause severe bleeding complications. In addition, the size of the residual kidney parenchyma is highly variable excluding the possibility of repetitive kidney biopsies during follow up. Therefore, we developed a new apparatus for standardized rat kidney biopsies to eliminate the apparent problems of rather traumatic and hardly standardized biopsy methods. We demonstrate that this new apparatus allows the implementation of two standardized and effective rat kidney biopsies within a single kidney without provoking secondary kidney damage or unwanted side effects that have to be considered for long-term outcome.

## Materials and Methods

### Experimental design

**Figure 1:**
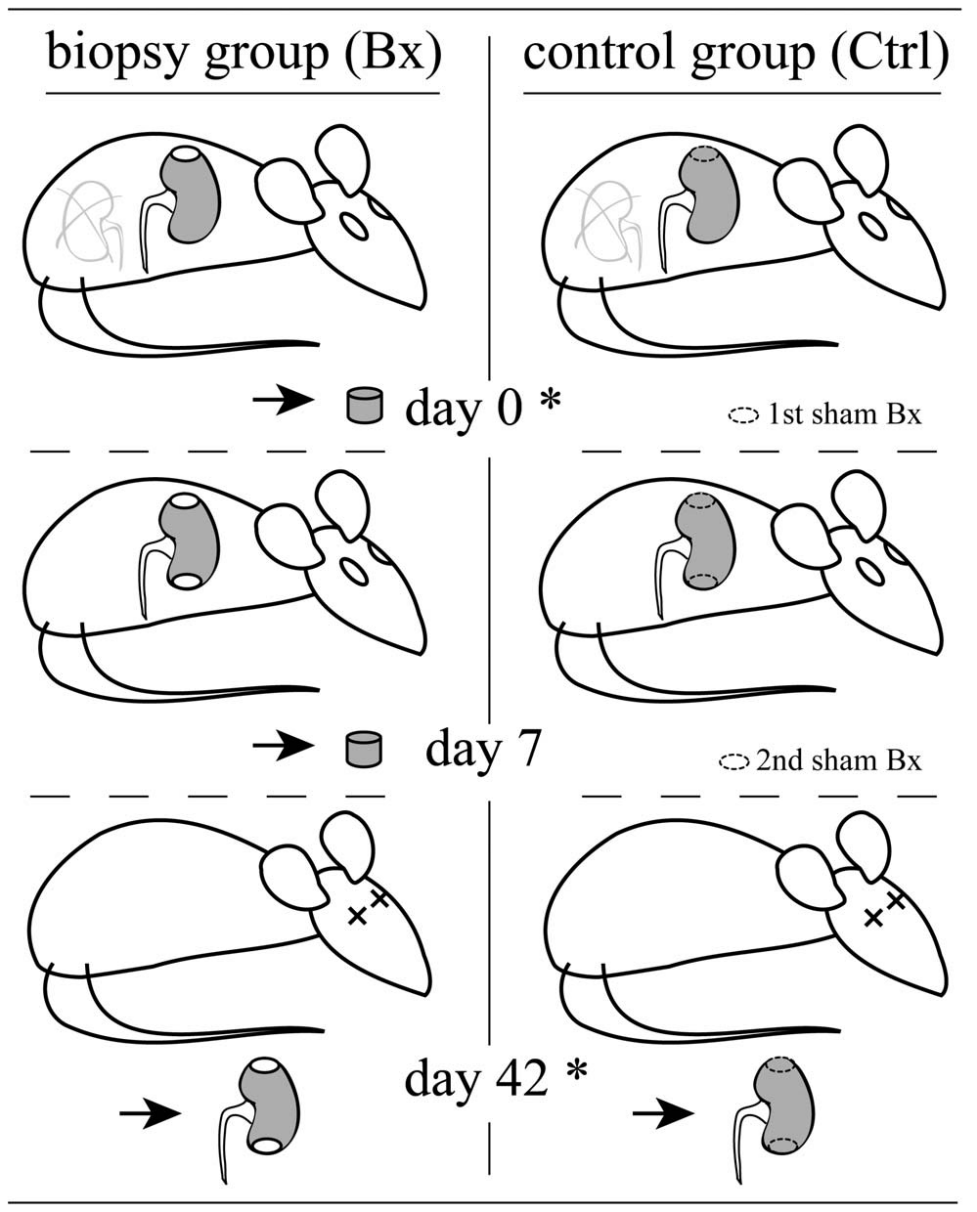
Experimental set-up. **Day 0**: Right nephrectomy in both groups. Subsequent biopsy of the upper pole of the residual kidney in the Bx group and sham biopsy of the residual kidney in the control group. **Day 7**: Biopsy of the lower kidney pole in the Bx group and second sham biopsy in the control group. **Day 42**: Animals were sacrificed and kidneys were removed for histology. Body weight, blood pressure, proteinuria and serum creatinine were measured before operations on day 0 and day 42 in both groups (*).

The animal study was carried out following the requirements of the National Act on the Use of Experimental Animals (Germany) and was approved by the University and State Animal Welfare Committees (Landesdirektion Sachsen, Referat 24: 24-9168.11-1/2010-48). 18 male Lewis rats (Charles River, Sulzfeld, Germany) were used for this study. The animals were fed standard rat chow (Altromin 1324, Spezialfutterwerke GmbH, Lage, Germany) and tap water ad libitum. The rats were randomized into two groups (Bx and Ctrl group, Fig. 1) and baseline values for body weight, blood pressure, proteinuria and serum creatinine were measured. The rats underwent surgery under general anesthesia with 1.5% to 2.0% isoflurane and all efforts were made to minimize suffering. On day 0, all rats underwent a right nephrectomy. Subsequently, the left kidney was gently exposed by the help of moist compresses and a 4 mm core biopsy was performed at the upper pole in the Bx group, whereas a sham procedure was performed in the control group. Kidney bleeding was stopped immediately by placing a preformed 4 mm Gelaspon patch (Chauvin Ankerpharm, Berlin, Germany) into the biopsy hole. The biopsy procedure was repeated on day 7 at the lower pole of the left kidney in the Bx group and a second sham operation was performed in the control group. Body weight, blood pressure, proteinuria and serum creatinine were measured again on day 42 in both groups. Animals were subsequently sacrificed and kidneys were removed for histology. Animal sacrifice was performed under general anesthesia with 1.5% to 2.0% isoflurane. After abdominal incision and removal of the left kidney a median thoracotomy with debridement of the great vessels was performed.

### Design of new biopsy apparatus

**Figure 2:**
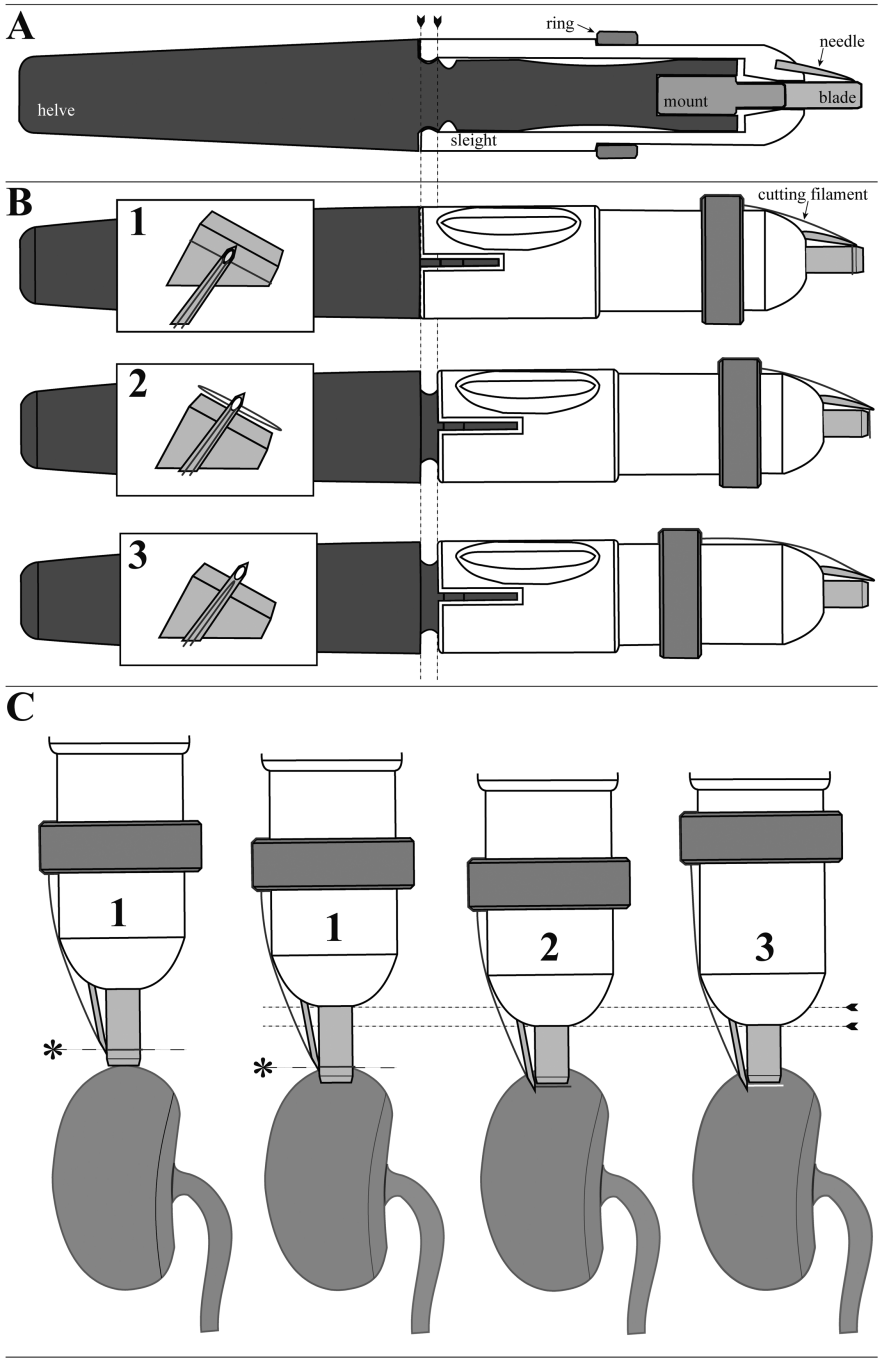
Technical drawing and operational principle of the new biopsy apparatus. **A**: Longitudinal section. The sleight (in white) and the helve (in dark grey) interact by a slot and key linkage where the tongue of the sleight being located inside snaps into the circular grooves of the helve (arrowheads). **B and C**: Operational steps 1–3. The cutting filament around the circular blade forms the reference point for the stitch depth (*).

The conceptual project design was executed by SolidWorks construction software (student version 2011). We combined the working principles of a biopsy punch and a cutting sling to minimize kidney damage during the biopsy process. Main parts of the biopsy apparatus are the helve and the sleight that interact by a slot and key linkage (Fig. 2 A). The helve bears a metal mount for exchangeable 4 mm core biopsy blades that were obtained from 4 mm skin biopsy punches (pfm medical ag, Cologne, Germany). The biopsy blades are made of stainless steel and can be used up to fifty times without a detectable loss of sharpness. The sleight carries a moveable ring on its corpus and a needle (0.75 mm diameter, stainless steel) at its top. Surgical suture material (Prolene 6–0, ETHICON, Norderstedt, Germany) served as cutting filament for cropping of the biopsy core. The helve, the sleight and the ring were formed by fused deposition modeling with a FDM Vantage S machine (Stratasys, Eden Prairie, USA) from acrylonitrile butadiene styrene (ABS) which is thermal stable from 85 to 100°C. Therefore the apparatus is not suitable for autoclaving. We use an aldehyde based chemical disinfection solution (gigasept FF, Schuelke & Mayr, Norderstedt, Germany) for cleaning of the apparatus after each use.

### Operational principle of new biopsy apparatus

**Figure 3:**
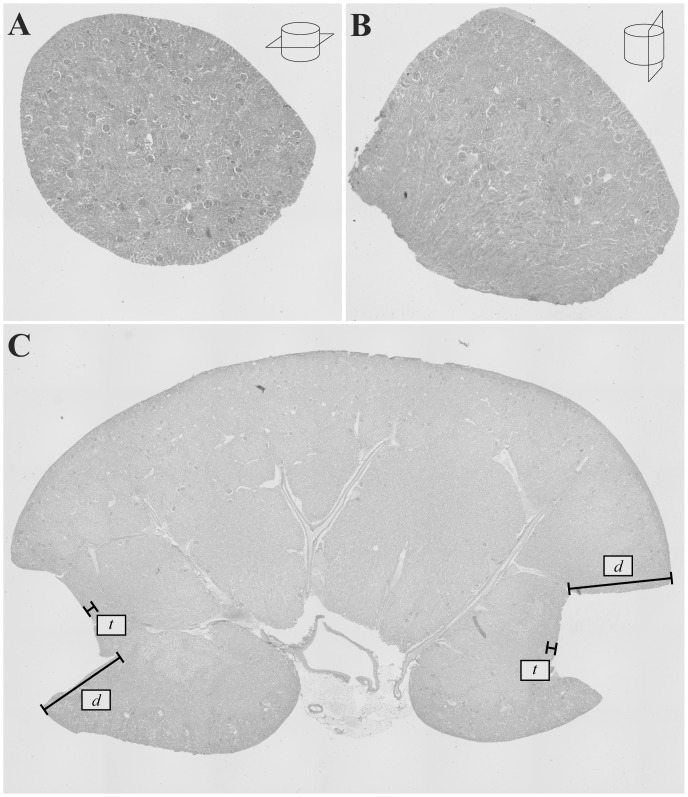
PAS staining. **A**: Transversal section of a 4 mm biopsy core with a mean of 57±28 glomerular cross sections per slice (magnification: ×4). **B**: Sagittal section (lateral cut) of a 4 mm biopsy core shows the whole cortical layer whereas the biopsy depth is limited to the region of the outer medulla (magnification: ×4). **C**: Longitudinal kidney cross section (wide-field composite image, figure-adjusted size). Biopsy depth *d* is indicated. Surgical tissue damage *t* is limited to the cortical area immediately adjacent to the taken biopsy cylinders.

The cutting filament has to be knotted to the ring. It has to be threaded through the eye of the needle and put around the circular blade to form a cutting sling (Fig. 2 B-1). The cutting sling around the circular blade forms the reference point for the stitch depth of the biopsy (Fig. 2 C-1). By fixing the helve with one hand, the sleight is pushed to its forward position and snaps into the second groove (Fig. 2 B-2). By this procedure, the cutting sling is shifted in front of the circular blade (Fig. 2 C-2). By pulling back the ring, the biopsy core is cropped at its base by the cutting sling (Fig. 2 B-3 and C-3).

### Histology

To investigate the biopsy depth as well as biopsy-related primary tissue damage, sagittal sections through both biopsy sites (day 0, day 7) were performed in all kidneys on day 42. Biopsy cylinders as well as dissected kidneys were fixed in methyl Carnoy's solution, embedded in paraffin and cut into 3 µm sections for periodic acid Schiff reagent (PAS) or indirect immunoperoxidase staining as described elsewhere [1], [2]. Indirect immunoperoxidase staining for ED-1 positive macrophages was performed with a murine monoclonal IgG_1_ antibody (AbD Serotec, Oxford, UK). Negative controls included either deleting the primary antibody or substitution of the primary antibody with equivalent concentrations of an irrelevant murine IgG_1_. The technical set-up included a Keyence BZ-9000 microscope (Keyence, Osaka, Japan). Digital image acquisition was performed with the built-in camera of the microscope. The digitalized images were stored in uncompressed TIFF format (1,360×1,024 pixels, 8 bit per color) for later analysis.

### Histological analysis of biopsy cylinders, biopsy depth and adjacent tissue damage

Images of PAS stained transversal biopsy cylinder sections on days 0 and 7 were obtained with an ×4 objective and the total number of glomerular cross sections per image was counted. Wide-field images of PAS stained sagittal kidney sections at day 42 were provided with the image stitching function of the BZ-II Analyzer software (x 20 objective). Biopsy depth and extent (maximum orthogonally to the biopsy bordering distance) of surgically damaged adjacent tissue (defined as PAS positive area of destroyed kidney architecture) was subsequently measured with the BZ-II Analyzer software.

### Renal morphology and counting of ED-1 positive macrophages

Images of PAS stained sagittal kidney sections were evaluated using well-established semi-quantitative scoring indexes (score: 0–4) for glomerulosclerosis and interstitial fibrosis as described elsewhere [3], [4]. Glomerular (x 40 objective, count: cells/50 glomeruli) as well as interstitial (x 20 objective, count: cells/mm^2^) ED-1 positive macrophages were counted as described elsewhere [5]. Images that showed surgical damaged tissue adjacent to the biopsy site were excluded from each analysis.

### Miscellaneous measurements

The weight of the rat body, the removed kidneys at day 0 as well as of the kidney biopsy cylinders was measured with a high precision scale (KERN, Balingen, Germany). Systolic blood pressure was measured noninvasively with a tail-cuff blood pressure recorder (Ugo Basile, Comerio, Italy). Proteinuria was measured by the pyrogallol red-molybdate complex method on an autoanalyzer (UniCel DxC 800 System, Beckman Coulter, CA, USA). Serum creatinine was measured using picric acid (Jaffé method, WAK-Chemie, Steinbach, Germany).

### Calculation of biopsy-associated reduction of functional renal mass

The 4 mm circular core biopsy blade has a 2 mm radius (*r*). Calculation of the biopsy cylinder volume *V*_Bx_ was conducted by the appropriate formula where *d* represents the mean biopsy depth (Fig. 3 C) and 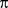 the constant defined by the ratio of a circle's circumference to its diameter. 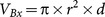



For calculation of the biopsy-associated loss of functional renal tissue-volume *V*_t_, the mean distance *t* (orthogonally to the biopsy, Fig. 3 C) of adjacent surgical tissue damage was added to *r* and *d*. 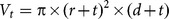



Finally, the biopsy-associated reduction of functional renal mass *m* was calculated with the accordant ratio equation where *BxW* represents the mean biopsy cylinder weight. 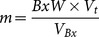



### Biometric planning and statistical analysis

The required number of animals was determined by means of a two-tailed *t*-test based on a Bonferroni-adjusted significance level. The main experimental target value (primary outcome) was serum creatinine. Body weight, blood pressure and proteinuria were secondary experimental target values. All numerical results are expressed as mean ±SD. After testing for normality with the Shapirow-Wilk normality test, statistical significance (defined as *P*<0.05) was evaluated using a Student's *t*-test.

## Results

### Renal biopsy procedure

We developed and used the new apparatus for rat kidney biopsy as demonstrated in Fig. 2 which is described in the methods section more detailed. Survival biopsies using this tool were performed under general anesthesia with 1.5% to 2.0% isoflurane. The mean value duration of each procedure amounted to 20 minutes.

### Analysis of functional parameters

At baseline, no differences with regard to body weight, blood pressure, serum creatinine or proteinuria were present in the 18 Lewis rats (data not shown). All rats having been biopsied (Bx group, n = 9) and the sham operated control group (Ctrl group, n = 9) survived without any peri-interventional complications such as infections or bleedings. On day 42, no statistically significant differences for body weight (388.50±22.61 grams, Bx vs. 375.00±21.21 grams, Ctrl, *P* = 0.38), blood pressure (135/80±35/15 mmHg, Bx vs. 130/80±30/15 mmHg, Ctrl, *P* = 0.65), proteinuria (0.58±0.15 mg/dl Bx vs. 0.55±0.07 mg/dl Ctrl, *P* = 0.76) and serum creatinine (35.10±9.57 µmol/l, Bx vs. 36.56±3.58 µmol/l, Ctrl, *P* = 0.57) were found between both groups (Table 1).

**Table 1:** Comparison of the biopsy group with the control group on day 42*.

	Category	Bx	Ctrl	*P* - value
Functional Parameters	Body Wt (grams)	388.50±22.61	375.00±21.21	0.38
	BP (mmHg)	135/80±35/15	130/80±30/15	0.65
	Proteinuria (mg/dl)	0.58±0.15	0.55±0.07	0.76
	SCr (µmol/l)	35.10±9.57	36.56±3.58	0.57
Morphologic Parameters	GS score	0.04±0.07	0.05±0.03	1.00
	IF score	0.00±0.00	0.00±0.00	n.a.
	Glom ED-1	5.94±1.48	5.93±1.18	0.88
	Inter ED-1	15.88±2.28	15.75±1.02	0.98

*Values are expressed as mean ±SD. Bx, biopsy group; Ctrl, control group; Body Wt, body weight; BP, blood pressure; Protein, proteinuria; SCr, serum creatinine; GS score, glomerulosclerosis score (see methods); IF score, interstitial fibrosis score (see methods); Glom ED-1, number of glomerular ED-1 positive macrophages/50 glomeruli; Inter ED-1, number of interstitial ED-1 positive macrophages/mm^2^; n.a., not applicable. Statistical significance was defined as *P*<0.05.

### Weight of kidneys and kidney biopsies

The mean weight of kidneys that were removed during unilateral nephrectomy on day 0 was 1220±110 mg (n = 18). Biopsy of the residual kidney with the new apparatus on day 0 and day 7 resulted in a constant specimen weight of 15±4 mg (n = 18) per biopsy cylinder.

### Histological analysis of biopsy cylinders, biopsy depth and adjacent tissue damage

To further investigate the biopsy quality, PAS staining of transversal sections of all biopsy cylinders was performed. Analysis demonstrated that a mean value of 57±28 (n = 18) glomeruli per transversal cylinder section (Fig. 3 A) was received. PAS staining of sagittal kidney sections and subsequent analysis of wide-field images verified that the biopsy depth of 1440±187 µm (n = 18) was strictly limited to the kidney cortex whereas the biopsy-related operative tissue damage (488±95 µm, n = 18) was limited to the cortical area immediately adjacent to the biopsy site (Fig. 3 C). For visual demonstration, a sagittal section of an additional biopsy cylinder was PAS stained (Fig. 3 B).

### Biopsy-associated reduction of functional renal mass

Calculation of biopsy-associated reduction of functional renal mass (consisting of biopsy cylinder and damaged adjacent tissue) resulted in a loss of 31.95 mg kidney tissue per biopsy. This corresponded to a relative renal mass reduction of 2.6% per biopsy when compared with the mean single kidney weight of 1220 mg on day 0.

### Renal morphology and counting of ED-1 positive macrophages

On day 42, semi-quantitative scoring (score: 0–4) for glomerulosclerosis (0.04±0.07 Bx vs. 0.05±0.03 Ctrl, *P* = 1.00) and interstitial fibrosis (0.00±0.00 Bx vs. 0.00±0.00 Ctrl) showed no differences between both groups (Table 1). No differences of ED-1 positive macrophage counts in glomeruli (5.94±1.48 cells/50 glomeruli Bx vs. 5.93±1.18 cells/50 glomeruli Ctrl, *P* = 0.88) as well as in the interstitium (15.88±2.28 cells/mm^2^ Bx vs. 15.75±1.02 cells/mm^2^ Ctrl, *P* = 0.98) were observed (Table 1).

## Discussion

To our knowledge, no apparatus for a standardized rat kidney biopsy has been introduced so far. Most research groups frequently use a partial (1/4 or 1/3) nephrectomy as survival biopsy procedure. This procedure cannot be highly standardized regarding the size of the biopsy which is especially critical in unilateral kidney disease models where a substantial loss of nephrons could also influence renal function by glomerular hyperfiltration with secondary acceleration of tubulointerstitial fibrosis [6]–[8]. Furthermore, the risk of fatal surgical or postoperative bleeding complications is a major barrier for the use of survival biopsies especially in time-consuming experimental setups such as renal transplantation or models requiring renal artery perfusion where a reduction of animal numbers would be especially desirable. To mimic the situation in single kidney disease models, we chose a study design where rats were unilateral nephrectomized before two consecutive biopsies of the residual kidney were performed. Two times biopsied rats lacked any peri-interventional complications and the analysis of functional parameters on day 42 showed no significant differences in regard to serum creatinine, proteinuria, blood pressure and body weight compared to the sham treated control group. Hereby, it needs to be considered that small changes of blood pressure may not be detected due to the relative high variability of the tail cuff blood pressure measurements. A mean value of 57±28 glomeruli per transversal section of the 4 mm biopsy core represented an adequate sample size for kidney histology whereas a biopsy depth of 1440±187 µm allowed to cut sufficient transversal sections by microtome. We demonstrate that the biopsy is strictly limited to the kidney cortex and that biopsy-associated operative tissue damage is restricted to the cortical area adjacent to the biopsy site. The reduction of functional renal mass was only 2.6% per biopsy and likely responsible for the low rate of side effects of this procedure. Further histological analysis of the kidneys biopsied twice and the control group on day 42 revealed that there was no difference in regard to glomerulosclerosis, interstitial fibrosis or ED-1 positive macrophage frequency between both groups. We would therefore conclude that our method is highly standardized and that even two sequential biopsies using this method (with a respective 5.2% reduction of vital kidney tissue) have no measurable side effects (as determined by analysis of respective functional and morphological parameters) in a single rat kidney.

Serum creatinine in a uni-nephrectomized model has already been a more sensitive marker of kidney function compared to any two-kidney models indicating a well-balanced ratio of biopsy size and residual kidney (even after two biopsies using this method). Nevertheless, since the glomerular filtration rate in this study was not measured by the more sensitive inulin clearance method, we cannot completely exclude slight alteration of the kidney function using our survival biopsy method. While the loss of 2.6% of renal tissue per biopsy is unlikely to be detectable in uni-nephrectomized rats, this influence in principle needs to be considered for studies using accelerated chronic progressive kidney disease models such as 5/6 nephrectomy or chronic allograft nephropathy. Nevertheless, in the situation of chronic progressive renal disease our new biopsy methodology demonstrates its special strengths due to small size and reproducibility. The possibility to perform a repeated biopsy in a single kidney allows for individualized, longitudinal evaluation of any therapeutic intervention by performing a zero biopsy (for baseline values) as well as a long-term follow up biopsy. In our view, it is most important that any potential influence of this kidney biopsy is as small as possible and at the same time highly standardized exerting a comparable effect to the interventional as well as to the corresponding non-treatment group.

While minimizing complications, the new device was able to provide kidney biopsy specimens of a standardized size that are entirely sufficient for various analyses in histology, biochemistry or molecular biology.

We conclude that the application of this new biopsy method is an animal and cost-saving approach for even single kidney disease models including renal transplantation in the rat that may further improve the statistical value of observational data due to the generation of related samples.
